# “We Can Do Anything but We Can't Do Everything”: Exploring the Perceived Impact of International Pediatric Programs on U.S. PICUs

**DOI:** 10.3389/fped.2019.00470

**Published:** 2019-11-15

**Authors:** Emily R. Berkman, Jonna D. Clark, Douglas S. Diekema, Mithya Lewis-Newby

**Affiliations:** ^1^Division of Pediatric Critical Care Medicine, Seattle Children's Hospital, University of Washington School of Medicine, Seattle, WA, United States; ^2^Treuman Katz Center for Pediatric Bioethics, Seattle Children's Research Institute, Seattle, WA, United States; ^3^Department of Bioethics and Humanities, University of Washington School of Medicine, Seattle, WA, United States; ^4^Division of Pediatric Emergency Medicine, Seattle Children's Hospital, University of Washington School of Medicine, Seattle, WA, United States; ^5^Division of Pediatric Critical Care Medicine, Section of Pediatric Cardiac Critical Care, Seattle Children's Hospital, University of Washington School of Medicine, Seattle, WA, United States

**Keywords:** international patients, pediatric intensive care units, resource allocation, health resources, United States, ethics, perceived impact

## Abstract

**Purpose:** Every year, an increasing number of international patients seek medical care in the United States (U.S.), yet little is known about their impact. Based on single institution experiences, we wanted to explore the perceived impact of international pediatric patients on large academic U.S. pediatric intensive care units (PICUs), as they are already taxed systems.

**Methods:** To explore current perceptions, seven geographically diverse institutions who advertise care for international patients on their websites and have ≥24 PICU beds were identified after IRB approval was obtained. We consented and interviewed PICU division chiefs or medical directors from each institution regarding their demographics and international patients. Common themes were identified.

**Results:** Participating institutions were diverse in geographic location, census, and resource allocation strategies. Five of the seven institutions reported the presence of a formal international patient program. Four of those five reported an increase in international patients receiving PICU care over the past 5 years. International patients sought complex surgeries, advanced cancer treatments and metabolic/genetic evaluations. We identified three primary domains that require further exploration and research: (1) cultural and language differences leading to barriers in providing optimal care to international patients (2) institutional financial considerations, and (3) perceived positive and negative impact on the care of local/domestic patient populations.

**Conclusions:** The presence of international programs raises a number of important ethical questions, including whether clinicians have a greater duty to serve residents of the local community as opposed to international patients when resources are limited. Further exploration is warranted.

## Introduction

*A family from another country brings their 7-year-old son to an academic pediatric institution in the United States for experimental chimeric antigen receptor T-cell therapy for refractory leukemia. During his hospitalization, he develops multi-organ failure requiring extracorporeal life support (ECLS). Unfortunately, he dies based on neurological criteria (“brain death”), but the embassy of his home country (the payer) refuses to allow discontinuation of ECLS*.

Every year, an increasing number of international patients seek medical care in the United States (U.S.) (1–3). International patients are often referred to as medical tourists or international medical travelers in the literature. Importantly, they are distinct from legal and illegal immigrants and refugees. However, the term “international patients” is quite broad and includes patients of all ages who are seeking care ranging from “routine” outpatient care to highly specialized inpatient care that is otherwise unavailable. Forms of payment for these services range from self-pay to government sponsorship (4–6). For the purpose of this paper, we narrowly define “international patients” as patients from other countries who seek inpatient care from an institution in the U.S. for a novel or otherwise unavailable therapy, who plan to return to their home country after treatment.

The challenges and opportunities associated with caring for international patients have been discussed in the literature (4, 5) and the press (6). However, these papers often refer to single institutional experience and do not separately examine outpatient and inpatient services (4–6). Moreover, they do not differentiate between pediatric and adult patients. Pediatric international patients represent a unique cohort with additional complexity that has not been formally evaluated to date. According to data from the U.S. Cooperative for International Patient Programs (USCIPP), increasing numbers of international *pediatric* patients are now seeking care in the U.S. Of the over 50,000 total international patients who sought care in the U.S. in 2015, children accounted for 52% of all international patients who required inpatient services (1).

Many pediatric patients come to the U.S. to participate in formal institution-based international patient programs that provide medical and surgical care unavailable in their home countries. The higher the complexity of the requested treatments, the greater the likelihood of pediatric intensive care unit (PICU) admission. Given additional baseline constraints on PICU resources (7, 8), we explored the perceived impact of international patients on U.S. PICUs.

## Methods

To explore areas requiring further investigation regarding the potential impact of international pediatric patients on PICUs at a multi-institutional level, we reached out to several U.S. pediatric academic institutions. Seattle Children's Hospital IRB approved the study under exempted status given the lack of identifiable information collected. Written consent was not required but verbal consent was obtained from the division chiefs or medical directors at the start of the interview. Selective sampling was used to identify seven geographically diverse academic, free-standing pediatric institutions who advertise care for international patients on their websites and have ≥24 PICU beds. The number of beds was used as a proxy for unit size, assuming that larger PICUs were more likely to have larger international patient populations. A PICU clinician and ethicist approached either a PICU division chief or PICU medical director from each institution. All institutions that were approached agreed to participate. Although other institutions would have met the aforementioned criteria, the participation of those initially approached provided geographic diversity for this pilot data.

Baseline demographic data, ICU staffing and capacity, catchment area, presence of other nearby PICUs, and distribution strategies between local PICUs were collected from each participant using a written survey. Semi-structured interviews were completed by phone or in-person to gather detailed information about PICU leaders' *perceptions* of the international patient population, including the presence/absence of a formal international program, countries represented, treatments sought, and payment type. Importantly, all answers were provided as *estimations*. Notes were taken during the interviews but were not recorded. When all the interviews were completed, notes were reviewed by the primary interviewer to identify common themes and questions requiring further exploration about potential impact of international patients on PICU care. These themes and questions were then vetted by the author group.

## Results

### Participant and Institutional Demographics

Four PICU division chiefs and three PICU medical directors participated from seven distinct sites. Number of PICU beds ranged from 24 to 55 and average daily patient census ranged from 20 to 55. Average annual PICU admissions ranged from 1,400 to 3,800 patients. ECLS capacity varied from three to eight circuits per institution.

Five of the seven respondents reported formal international programs. Four of the seven reported an increase in international patients receiving PICU care over the past 5 years. Two observed a sizeable presence for ≥10 years. One reported only an occasional international patient requiring PICU admission. The *estimated* total number of international patients ranged greatly. One institution reported only one international patient requiring PICU admission in the past year whereas another institution reported ~3–8 international patients requiring PICU care per week. Patients represented many countries. Respondents identified complex surgeries, advanced cancer treatments and metabolic/genetic evaluations as common reasons for seeking international medical care (see Table 1).

**Table 1:** PICU demographic information, international patient characteristics and perceived impact by institution.

**Institution**	**1**	**2**	**3**	**4**	**5**	**6**	**7**
U.S. Region	Northwest	Southwest	South Central	Midwest	Midwest	Northeast	Southeast
Additional PICU within 50 miles	Yes	Yes	Yes	No	Yes	Yes	Yes
International patient presence	>10 year, increase last 2–3 years	>10 year	Increase last 3–5 year	Minimal	>20 year, increase last 5 years	Increase last 5–10 years	>20 year
Countries represented	ME, Europe, China	ME, China	ME, Mexico	Brazil	ME, Japan	ME, Europe, South America	ME, Bahamas
Reason for seeking medical care	Immunotherapies, complex surgeries	Cancer, complex surgeries, metabolic/genetics	End-stage pulmonary disease, neurodegenerative disorders	Complex surgeries	Immunotherapies, complex surgeries	Immunotherapies, complex surgeries, metabolic/genetics	Novel therapies, complex surgeries, metabolic /genetics
Payment	Govt., Self-pay, charity	Govt.	Govt., self-pay, charity	Hospital	Govt, self-pay, charity	Govt, self-pay, charity	Self-pay or insurance
Perceived effect on PICU	Yes	No	Yes	No	Yes	Yes	No
Perceived effect on local population	Maybe	No	Yes, improved cultural competence	No	No	No, but strain on system	No
Perceived change in PICU medical care provided	No	No	No	No	No	Yes	No
Cultural training occurring	No	No	No	No	No	Yes	No
Cultural conflict present	Yes	No	Yes	Yes	Yes	Yes	No

*ME, Middle East, Govt., Home government sponsored*.

### Domains of Perceived Potential Impact

We identified three domains where clinicians perceived a potential impact of international programs on pediatric critical care: (1) cultural and language differences leading to potential barriers in providing optimal care to international patients, (2) institutional financial considerations, and (3) impact on the care of local/domestic patient populations (see Table 2).

**Table 2:** Example quotes regarding the impact of international patients on the delivery of pediatric critical care.

**Themes**	**Quotes**		
Perceived impact on PICUs and local Population	“One of the reasons our international referrals have not impacted resources is that we have already been at, or over capacity. I have to deny far more than I can accept. I've been vigilant to only accept those who will benefit from subspecialty care…I screen so heavily because I play the census game locally.”	“There is also concern that international patients are very demanding. We sometimes have double occupancies during high census. Sometimes we don't want to battle with the international families, but we don't even ask the domestic families. We just double them up. It doesn't affect the care but sometimes choices made for the sake of the system are not equally distributed across international and domestic groups.”	“When ICU capacity is limited, it potentially limits access to our own patients in the neighborhood and region. It is something we should think about with the ongoing growth of the international program.”
Cultural differences	“I think that international patients have differing expectations. Their expectations are often not aligned with reality. This more commonly leads to issues with trust and communication than culture does. There is this magical thinking.”	“Families are very stressed from the “newness” of the situation, are far removed from their homes, family members and communities. They are very isolated and this makes everything more challenging. Then you add the stress of them being in the ICU. It results in great stress for both families and providers.”	“Yes, most common is around end of life issues and/or limiting care. Many families can't even conceive of these issues. ‘It’s in god's hands' is a common fall back. It has caused a lot of problems.”
Finance	“At the end of the day it comes down to money. There are huge profit margins for these patients. Eighty percentage of our ICU is on Medicaid so the international patients help with costs a lot.”	“At the end of the day international patients are extremely desired because of the financial piece. But the sustainability of the program is a challenge… PICU's are caught in the middle and end up providing the most expensive part of the care. As a result, the PICU absorbs the extra costs. To prevent this we need to have good cost estimates, get the right patients and keep costs down.”	“I have more of a conflict when we are actively seeking patients out to come to us, knowing that some of that reason is financial in origin. It feels slimy…When money gets involved, it's a little insincere.”
ECLS	“We had Saudi patient and there were challenges with end of life communication (patient was on ECLS). We had to go through the embassy. Our experience will inform future contracts.”	“A patient came for a lung transplant but clinically declined and required ECMO…if the embassy found out that parents had assented to withdrawal of support they would be in legal trouble once they got home. The embassy was not willing to provide assent.”	“We, the medical team, were all in agreement that ECMO was futile but the family was not in agreement. We finally came to an agreement that if the circuit went down we wouldn't replace it and the family was able to see that as god saying it was her time. We have now changed our ECMO consent to include that if the medical team deems the support to be futile, it will be stopped.”
Duty	“As healthcare providers on the frontline we do and should take care of the patients in front of us. However, this is within the limits or our resources and with an appropriate system of reimbursement. This is true as long as it doesn't impact the care of the local community.”	“I think we do. But there are some realistic challenges and limits that we need to be aware of, especially around end of life care. I'm not sure everything we do in the ICU has its place in the global venue. Not everyone needs to die on ECMO or after their 10th experimental chemo run for refractory cancer.”	“I like the quote ‘We can do anything but we can’t do everything'. We need some way of defining the limits of what we can provide.”

#### Cultural Differences

Respondents expressed concern that cultural differences potentially affect the quality of care for international patients in the PICU. All institutions have interpreter services but only one institution has a formal training in cultural diversity for staff. Importantly, respondents observed that although many domestic patients come from diverse cultures, cultural differences among international patients are magnified in the absence of local community support. Additionally, some respondents perceived that international patients/families often had different understandings and expectations about what is possible in U.S. hospitals. Since five of the international programs offer experimental treatments for life-threatening illnesses that failed conventional therapy, some international patients die in U.S. hospitals. Respondents expressed that differences in end-of-life cultural norms can lead to conflict between clinicians and families. Specifically, three institutions experienced challenges discontinuing ECLS for patients with irreversible diseases. One institution changed its consent form to explicitly permit the medical team to discontinue ECLS therapy when continuation is deemed inappropriate.

#### Finance

Although not included in any of international program mission statements, four respondents expressed concern that institutional financial incentives play a role in recruiting international patients. Respondents perceived that most international patients are financed through government sponsorship or self-pay, and worried that this approach may be more profitable compared to private insurance or Medicaid. However, one respondent expressed concern that unanticipated PICU stays often yield additional expenses, resulting in financial deficits for the institution.

#### Impacts on Local/Domestic Populations

Four out of seven respondents reported perceptions that international patients impact the PICU. However, only one respondent perceived a direct impact on the local population, which was improved cultural humility. Five respondents worried about how international patients might impact their future ability to care for the local population given anticipated programmatic growth. One respondent reported that despite the international program growth, no additional PICU resources were added. Several respondents perceived that international patients requiring PICU admission had long lengths of stay and required additional resources to navigate cultural and language differences. One respondent reported that hospital admission for international patients is often declined due to continuous lack of PICU capacity. Another institution accepts international patients even when resources are limited and uses double room occupancy. This respondent observed that local families seemed more likely to be placed in double rooms because of the perception that international families do not find room sharing acceptable.

## Discussion

The seven participating institutions were diverse with respect to their geographic location, census, size of the international programs, and resource allocation strategies. While we recognize the small sample size and lack of representation of international patient program directors, the responses of the clinicians to our questions highlight a number of important ethical dimensions that require further exploration. We also acknowledge that providing incredibly valuable and potentially lifesaving therapies to children who lack access in their home countries is very important, and therefore understanding how to optimize outcomes for these programs and how they impact the care of the local/domestic population is crucial.

The concerns raised by the clinicians regarding the potential impact of cultural differences on the quality of medical care provided, highlight the importance of further exploration and investigation into how international patient programs assess potential cultural differences prior to accepting new patients. Addressing these differences a priori may reduce cultural conflict during the hospital stay. For example, predictable cultural conflicts such as those surrounding end of life care, already exist within our diverse domestic population. On a daily basis in the ICU setting, we often grapple with how we can best honor the values of patients and families when their belief systems differ significantly from our own. In caring for the international population, cultural conflicts may be further intensified. Arguably, the candidates who are best served by program enrollment are those international patients who are most likely to benefit from a requested treatment that is not otherwise available to them. However, what if the risk for mortality is >80%, even if one receives the experimental treatment? Many families in this situation are so hopeful for survival that they are not able to comprehend the high risk of death, especially in a geographic location far from their family and local community. In these cases, what preparation and counseling should be offered to these families in anticipation of the possibility of a very poor outcome? Furthermore, the potential lack of exposure to Western medical practice opens the doors to additional conflict. For example, in some countries, unlike the U.S., “brain death,” or death by neurological criteria, is not accepted as equivalent to death, legally or medically (9). To address potential conflict and clarify expectations, an explicit discussion regarding the consequences of potential failure of therapy and the medical and legal definitions of death in the U.S. needs to occur prior to acceptance into the international program.

These conversations may need to extend beyond the immediate family. In cases where government sponsorship is instituted, exploring what conversations should occur with the embassies prior to acceptance into the program is important. Preemptively modifying consent for care forms, and being explicit regarding cultural practices in the U.S. is critical. While respect for patient and family autonomy is the predominant model for medical decision-making in the U.S., not all cultures align with this model. Sometimes extended family members, religious leaders, or even the government may have the authority to make decisions, rather than the individual patient and family (10). Exploring and understanding how to work through modified decision-making models is also essential prior to acceptance. Finally, once accepted into the program, incorporating cultural navigators and language interpreters, in addition to providing training in cultural humility for all staff will benefit *all* patients and their families.

The financial questions raised by our participants similarly demonstrate the need for programmatic transparency. Importantly, knowledge of how international patients are expected to pay and how the generated revenue is distributed is necessary. Non-for-profit U.S. hospitals still require revenue in excess of cost. If international programs help maintain institutional financial stability, then all patients seeking care at the institution may benefit. Notably, some children within local communities lack adequate insurance coverage and would benefit from increased uncompensated care funds. Ideally, if revenue is generated from these programs, the monies could be directly used to serve those in need, both locally and internationally. Furthermore, potentially subsidizing international programs to maintain equity in access for all international patients, not just for those who can pay or are sponsored by the government, also may be important. As providing care for all international patients is too far reaching, investment in sustainable global health programs may be a way of reaching more patients in their home countries.

Finally, five out of seven of our participants also expressed questions about the potential future impact of international patients and programs on limited PICU resources. As pediatric international programs grow, there is risk that the number of patients will outpace programmatic infrastructure resulting in further stress on already taxed PICU systems. While acceptance of international patients does not directly equate to denying local patients access, placing additional strain on a resource-limited system may potentially risk limited access for local/domestic patients. In this setting, do tertiary care pediatric institutions have a greater obligation to provide care to *all* children or should children in the local community be prioritized? In general, if resources are limited, how should international programs be instituted so that the local/domestic community does not have reduced opportunities to access local medical care and services? Although ideally the goal of equitable access to medical care is often on the global scale (11), the current reality of resource scarcity and unequal distribution of access to health care within individual countries makes achieving this on a global level very difficult. In addition, special duties owed to others based on specific relationships is also important to consider (12). For example, U.S. taxpayers subsidize physician education and training through the federally funded programs of Medicare and Medicaid. Therefore, one may argue that U.S. physicians have a greater duty to prioritize providing medical care to local/domestic children. Lastly, the complex and often experimental treatments typically sought by international patients are often beyond the minimal threshold meeting basic healthcare needs (13). Hence, it is unacceptable if the provision of complex and/or experimental medical care of an international patient directly prevents the provision of a basic level of care for a local child. Although differential access to experimental or novel therapies within the home country demonstrates clear inequality, those without access to a sufficient level of basic care represent greater injustice (13). As one respondent stated, “I'm not sure everything we do in the ICU has its place in the global venue. Not everyone needs to die on ECMO (ECLS) or after their 10th experimental chemo run for refractory cancer.” Identifying the proper balance of caring for both the domestic and international populations is the ethical challenge. As another participant pointed out, “If we accepted every international patient who wanted care from the United States, our healthcare system would break.” So where should we draw the line?

## Limitations

There are a number of important limitations to this exploratory study. First, the sample size is small and includes only those institutions we identified, allowing for potential selection bias. Second, we only collected the opinions of PICU clinicians. Their perceptions may not reflect those in other roles. The true impact of these programs requires more diverse and extensive exploration of perceptions. The addition of quantitative data regarding the number of international patients in the PICU, their lengths of stay, billable hours spent on their care, etc. would also be important to analyze. A more encompassing assessment would provide a clearer picture and help institutions to provide the best care possible for all patients.

## Conclusion

Our observations suggest a growing practice of accepting and recruiting international patients to U.S. pediatric institutions. The questions raised by this preliminary exploration demonstrate the necessity of additional examination and investigation into the complexities of international patient programs. Respondents raised questions about the impact of cultural and language differences on the quality of care provided to these international patients. They also raised concerns regarding the potential impact of these programs on pediatric critical care delivery for local and domestic populations. As pediatric international patient programs continue to expand, understanding the financial impacts of these programs is important. Furthermore, developing systems to promote the most equitable and just distribution of limited health care resources amongst all domestic and international patients is crucial. Ultimately, through further study, creative systems may be developed and created within international patient programs in order to provide the highest quality of equitable healthcare for all children.

## Data Availability Statement

The datasets generated for this study (de-identified) are available on request to the corresponding author.

## Author Contributions

EB conceptualized the pilot study, conducted the survey and interviews, performed the initial analysis, drafted the initial manuscript, and approved the final manuscript as submitted. JC, DD, and ML-N helped to design the study questions, vetted themes and questions, reviewed and revised the manuscript, and approved the final manuscript as submitted.

### Conflict of Interest

The authors declare that the research was conducted in the absence of any commercial or financial relationships that could be construed as a potential conflict of interest.
